# Gastric adenocarcinoma is not an HIV related malignancy

**DOI:** 10.1186/1742-4690-9-S1-P139

**Published:** 2012-05-25

**Authors:** Violet Kayamba, Akwi Asombang, Mpala Mwanza, Edford Sinkala

**Affiliations:** 1 Tropical Gastroenterology and Nutrition Group, University of Zambia School of Medicine, Zambia; 2 Washington University School of Medicine in St Louis, Missouri, USA

## Introduction

HIV infection has been shown to increase the risk of developing some malignancies. We evaluated the possibility of an association between gastric cancer and HIV infection in patients seen at the University Teaching Hospital, Lusaka, Zambia. Other known risk factors such as infection with Helicobacter pylori (H. Pylori), presence of CagA, serum pepsinogen 1 to 2 ratios, smoking and alcohol intake were also evaluated.

## Methods

This was a prospective case-control study with cases being patients with gastric adenocarcinoma confirmed by histopathology while controls were patients without visible mucosal abnormality. Two controls were enrolled for each case after matching for age and sex. The presence of HIV and H.pylori antibodies, the virulence factor CagA and serum pepsinogen 1 and 2 levels were determined using ELISA. Odds ratios were calculated to determine the presence of any association. Results were analysed using STATA 10.

## Results

A total of 38 cases and 76 controls were enrolled. There was no association between gastric cancer and HIV infection (Odds Ratio 1.41, 95% CI 0.3-6.4; P=0.73). Smoking and alcohol were found to increase the odds of developing gastric cancer, with P values after multivariate logistic regression of 0.04 and 0.02 and odds ratios of 3.5 and 3.1 respectively. Overall, 81% of the patients were found to be positive for H. pylori infection, with no significant difference between the cases and the controls (P=0.24). The presence of antibodies to CagA was also not different between the two groups (P=0.79). Serum levels of pepsinogen 1 were not significantly different between the two groups (P=0.45). However, the presence of a low pepsinogen 1 to 2 ratio was more discriminating, with a higher proportion among the cases (P=0.009).

## Conclusions

No association was found between gastric cancer and HIV infection. Alcohol and smoking increase the odds of developing gastric cancer. Patients with gastric cancer have lower pepsinogen 1 to 2 ratios, although there is no difference in the levels of pepsinogen 1, H.pylori infection and CagA between gastric cancer patients and healthy controls.

